# Can a weekend of controlled hypoxia restore hormonal balance? A novel approach to stress recovery in aviation professionals

**DOI:** 10.3389/fphys.2025.1582591

**Published:** 2025-06-26

**Authors:** Jose Francisco Tornero-Aguilera, Francisco José Martin-Gomez, Manuel Martinez-Taranilla, Alejandro Rubio-Zarapuz, Alexandra Martín‐ Rodríguez, Vicente Javier Clemente-Suárez

**Affiliations:** ^1^ Graduate School of Business, Universidad ESAN, Santiago de Surco, Lima, Peru; ^2^ Department of Sports Sciences, Faculty of Medicine, Health and Sports, Universidad Europea de Madrid, Madrid, Spain; ^3^ Faculty of Health Sciences, UNIE, Madrid, Spain; ^4^ Grupo de Investigación en Cultura, Educación y Sociedad, Universidad de la Costa, Barranquilla, Colombia

**Keywords:** normobaric hypoxia, stress, aviation professionals, HPA axis, prolactin, cortisol, testosterone, DHEA-S

## Abstract

**Background:**

Aviation professionals experience chronic stress due to prolonged work hours, irregular schedules, and exposure to altitude-related physiological challenges, leading to dysregulation of the hypothalamic-pituitary-adrenal (HPA) axis. Controlled hypoxia has been proposed as a potential strategy for modulating stress responses and enhancing recovery.

**Objective:**

This study aimed to investigate the effects of a weekend of controlled normobaric hypoxia on key hormonal markers, including cortisol, prolactin, testosterone, and DHEA-S, in pilots and flight attendants.

**Methods:**

A within-subject experimental design was used with 10 participants (6 pilots, 4 flight attendants) exposed to two 12-hour sessions of normobaric hypoxia (simulated altitude: 3,500–4,000 m) during their designated rest period. Blood samples were collected at three-time points: baseline, pre-hypoxia, and post-hypoxia, and analyzed for hormonal changes.

**Results:**

Prolactin levels significantly increased post-intervention, indicating a neuroendocrine stress response. Cortisol levels showed a moderate decrease, suggesting potential stress adaptation. Testosterone levels significantly increased in both groups, supporting anabolic recovery mechanisms. DHEA-S exhibited a slight but non-significant increase, while the DHEA-S/cortisol ratio improved, suggesting enhanced stress resilience.

**Conclusion:**

Short-term exposure to normobaric hypoxia induced significant hormonal adaptations, potentially aiding stress recovery in aviation professionals. These findings highlight the potential of controlled hypoxia as a non-pharmacological intervention for stress management in high-demand professions. Further research is needed to refine hypoxia protocols and assess long-term effects.

## 1 Introduction

The physiological and psychological effects of hypoxia have been thoroughly examined in both controlled laboratory conditions and actual high-altitude surroundings (Zhang et al., 2022; Ramírez-delaCruz et al., 2024). Hypoxia, defined by diminished oxygen availability, triggers a series of adaptive reactions to preserve equilibrium. These adaptations encompass enhanced erythropoiesis, respiratory changes, and neuroendocrine alterations. Hypobaric hypoxia (HH) occurs due to reduced atmospheric pressure, leading to lower oxygen partial pressure, as seen in high-altitude environments or hypobaric chambers. In contrast, normobaric hypoxia (NH) maintains normal atmospheric pressure but reduces oxygen concentration, typically using hypoxic rooms or masks (Rosales et al., 2022). HH induces more pronounced hyperventilation and dehydration, while NH provides a controlled environment for hypoxia exposure. HH is commonly used for altitude training and acclimatization studies, whereas NH is applied in intermittent hypoxia therapy and performance enhancement (Millet et al., 2010). While both hypobaric and normobaric hypoxia elicit similar physiological responses—such as increased ventilatory drive, oxidative stress, and hormonal changes—their application contexts differ significantly. Hypobaric hypoxia, associated with high-altitude exposure or flight simulation chambers, is often linked to environmental or operational stressors. In contrast, normobaric hypoxia provides a controlled, replicable, and safer model that allows for targeted interventions without the confounding effects of barometric pressure changes. This makes NH particularly suitable for studying and managing stress-related physiological adaptations in occupational settings such as aviation, where safety, replicability, and low-risk implementation are essential. Although extended or intense hypoxia can adversely affect cognitive and physical performance (Komiyama et al., 2017), regulated exposure to mild or intermittent hypoxia has been proposed as a potential therapeutic method for improving physiological resilience and stress recovery (Timon, Martinez-Guardado, and Brocherie, 2023).

In occupational and high-performance environments, such as aviation, military, and sports, individuals are often subjected to stressors that disrupt their homeostatic balance (O’hara et al., 2022; Suarez et al., 2018; Redondo-Flórez et al., 2020). Extended work hours constitute a significant factor contributing to chronic fatigue and burnout among these stressors. Due to the inadequate recovery periods disrupt circadian rhythms (Nobari et al., 2023), diminish cognitive function, and result in cumulative weariness, hence heightening the risk of decision-making errors, mood disorders, and long-term health issues. Studies indicate that individuals involved in shift work or irregular schedules frequently have disrupted sleep patterns and an increased prevalence of metabolic and cardiovascular diseases, hence intensifying the physiological strain (Li et al., 2024).

The hypothalamic-pituitary-adrenal (HPA) axis is pivotal in regulating this body’s stress response. Prolonged exposure to occupational stressors, including extended work hours and elevated cognitive demands such as pilots and fly attendants, results in overactivation of the HPA axis, causing persistent cortisol secretion (Hinds and Sanchez, 2022). Consequently, the persistent elevation of cortisol levels correlates with compromised glucose metabolism, diminished immunological function, and heightened susceptibility to psychiatric illnesses, such as anxiety and depression. Furthermore, disruption of the HPA axis has been associated with chronic fatigue, reduced motivation, and the emergence of burnout syndrome, which is defined by emotional weariness, depersonalization, and a decline in personal achievement (Tanriverdi et al., 2007). Also in this regard, burnout is typically suffered by this kind of individuals, complicated with serious consequences for one’s emotional and physical health such as the progressive drain of psychological resources caused by occupational stress and excessive workload. Disruptions to sleep, anger, cognitive impairments, and chronic fatigue are common signs of burnout. Related to this, it is needed to develop effective treatments to reduce the consequences of burnout because it is associated with neuroendocrine abnormalities like chronic HPA axis activation and disturbances in stress hormones (Salvagioni et al., 2017).

Recent studies have emphasized the efficacy of controlled hypoxia in regulating stress-related hormonal responses (Allsopp et al., 2024). Researchers have investigated the impact of hypoxia-induced adaptation in facilitating recovery, optimizing neuroendocrine balance, and boosting autonomic regulation. Although, exposure to hypoxia can lead to various alterations in the nervous system, including acute and chronic mountain sickness, memory loss, and cerebral edema (Liu et al., 2024). Its involvement in the pathogenesis of depression has also been suggested, Kushwah and collaborators pointed out that intermittent hypobaric hypoxia (IHH) may have a neuroprotective effect (Kushwah et al., 2016). However, short-term normobaric hypoxia, increasingly used in stress-relief programs, was tested in management professionals to assess its effects on stress hormones. Results showed increased prolactin and cortisol levels, along with a decreased DHEA-S/cortisol ratio, indicating a significant stress response rather than the expected relief (Ruzic et al., 2024). Nevertheless, the majority of research has concentrated on altitude adaptation or intermittent hypoxia training within sports cohorts (Filopoulos, Cormack, and Whyte, 2017), resulting in a deficiency of comprehension regarding its prospective applicability for those subjected to persistent occupational stress (Coppel et al., 2015). Also, normobaric hypoxia has become an innovative non-pharmacological therapy to treat cognitive dysfunction. Nevertheless, the acute effects of exposure to hypoxia on cognitive performance remain unclear (Ramírez-delaCruz et al., 2025). Aviation professionals represent an ideal population for hypoxia-based interventions due to their frequent exposure to mild hypoxic conditions during flight and their heightened vulnerability to cumulative occupational stress. Pilots and flight attendants often operate under cabin-pressurized environments equivalent to 1,800–2,400 m of altitude, which, combined with sleep disruptions, irregular schedules, and high cognitive demands, can lead to chronic stress and neuroendocrine dysregulation. Given their familiarity with low-oxygen conditions and the logistical constraints of their work-rest cycles, this population is well-suited for testing the effectiveness of non-invasive normobaric hypoxia protocols designed to enhance recovery and stress resilience.

In consequence, this study aimed to investigate the effects of a weekend of controlled normobaric hypoxia on key hormonal markers, including cortisol, prolactin, testosterone, and DHEA-S, in pilots and flight attendants. Our findings may offer significant insights into the practical uses of hypoxia-based therapies for improving resilience and recovery in high-stress conditions.

## 2 Materials and methods

### 2.1 Study design

This study utilized a within-subject experimental design to investigate the effects of short-term normobaric hypoxiaon the hormonal stress response of private aviation pilots and flight attendants. Unlike previous studies conducted in enclosed altitude chambers, which may induce discomfort and disrupt sleep quality, this study implemented a hypoxia exposure protocol using a comfortable, ergonomically designed mask system. This minimized participant burdenand ensured high compliance while allowing for a more natural restorative sleep environment.

The study was conducted over a single weekend, coinciding with the mandatory rest period of participants following a series of long-haul flights. The intervention aimed to replicate the mild hypoxic conditions encountered during high-altitude flight operations while ensuring an optimized recovery environment. This approach enabled researchers to evaluate the endocrine stress response in a real-world aviation setting, without the confounding effects of acute flight-related fatigue or restricted movement.

Each participant served as their own control, allowing for direct comparisons between their baseline condition (during their regular workweek) and their hypoxic intervention condition (during the weekend rest period).

To control for external confounding factors, participants:

• Maintained consistent dietary intake and hydration levels for at least 3 days prior to the study.
• Avoided strenuous exercise or any physical activity exceeding the anaerobic threshold throughout the study period. To objectively monitor compliance with physical activity restrictions, all participants wore wrist-worn fitness trackers (Garmin® or Polar® devices), which recorded step count, movement intensity, and estimated energy expenditure throughout the intervention period. Activity data were reviewed daily by the research team to ensure that no participant exceeded light-to-moderate physical activity thresholds. Additionally, participants completed daily digital checklists confirming adherence to study instructions, including rest, diet, and sleep routines.
• Were monitored for psychosocial interactions, ensuring similar exposure to work-related or social stressors during both conditions.
• Completed pre-study interviews to confirm compliance with protocol requirements.

Additionally, participants performed brief motor coordination tests at three distinct time points to assess potential neuromuscular effects of hypoxia exposure. These sub-minute tests were conducted 1 hour prior to blood sampling to ensure results were not influenced by transient fluctuations in autonomic activity.

All participants provided written informed consent, and the study was conducted in accordance with ethical standards approved by the Institutional Review Board (Approval Code: NUMA-2024-012).

### 2.2 Participants

A total of 10 private aviation professionals (six male pilots and four female flight attendants) voluntarily participated in the study. The mean age of participants was 38.4 ± 6.8 years. All individuals were actively employed in private executive aviation and routinely operated long-haul intercontinental routes. All female participants were in the pre-menopausal period, as confirmed by self-reported menstrual regularity and absence of hormonal treatment.

Eligibility criteria required participants to:

• Be between 30 and 50 years old.
• Hold a valid aviation medical certificate.
• Have accumulated at least 1,000 flight hours.
• Be free from cardiovascular, respiratory, or endocrine disorders.
• Not have undergone hypoxia or altitude training in the past 6 months.

Exclusion criteria included smoking, history of hypertension, sleep disorders, recent participation in endurance competitions, or use of pharmacological stimulants affecting stress hormone regulation.

Throughout the study, participants adhered to their standard sleep-wake cycle. Daily activity levels were monitored via wearable fitness trackers, ensuring no significant deviation from their usual movement patterns. The mean step count remained stable at 8,300 ± 1,250 steps/day, confirming that external lifestyle factors did not interfere with the intervention.

### 2.3 Procedure

Before The hypoxia intervention was conducted over the weekend, utilizing the MITOVIC® hypoxia training system, a specialized device developed by COMMIT GmbH (Salzgitter, Germany). The MITOVIC® system enables precise modulation of inspired oxygen fraction (FiO_2_), simulating altitudes ranging from 2,000 to 5,000 m (FiO_2_ 16.5%–13.5%).

Key advantages of the MITOVIC® system over traditional hypoxia chambers include:

Enhanced sleep quality: Participants remained in their usual sleeping environments rather than enclosed chambers, reducing disruptions in circadian rhythm.

Increased comfort: The low-profile ergonomic mask was designed to ensure unrestricted movement and minimal discomfort during sleep.

Adaptive oxygen control: The FiO_2_ levels were dynamically adjusted based on real-time physiological feedback, preventing excessive hypoxemia.

### 2.4 Intervention protocol

Participants were exposed to normobaric hypoxia for two consecutive nights under the following conditions:

• Duration: Two 12-hour sessions (8:00 p.m. to 8:00 a.m. each night).
• Simulated Altitude: 3,500–4,000 m (FiO_2_ ∼14.5%).
• Environmental Setting: Participants slept in their usual bedroom environments while using the MITOVIC® mask system.

Peripheral oxygen saturation (SpO_2_) was continuously monitored throughout the hypoxia exposure, with specific measurements taken at the following time points:

1. One hour after initiating hypoxia exposure.
2. Immediately before sleep onset.
3. Upon waking up in the morning.

SpO_2_ levels were recorded using Beurer Po45 pulse oximeters (Beurer GmbH, Ulm, Germany) and analyzed to determine individual hypoxic adaptation profiles.

#### 2.4.1 Hormonal assessments

To evaluate the endocrine stress response, blood samples were collected at three distinct time points:

1. Baseline (Wednesday, 48 h before intervention).
2. Pre-hypoxia (Friday morning, before initiating hypoxia exposure).
3. Post-hypoxia (Sunday morning, immediately following the intervention).

All blood samples were drawn at 9:00 a.m. to account for diurnal hormone fluctuations, following an overnight fastand 30-minute rest period in a dimly lit, temperature-controlled environment.

The primary hormonal biomarkers analyzed included:

• Cortisol–Marker of hypothalamic-pituitary-adrenal (HPA) axis activity and stress response.
• Prolactin–Associated with fatigue, neuroendocrine adaptation, and physiological stress.
• Dehydroepiandrosterone-sulfate (DHEA-S) – A counter-regulator of cortisol, linked to stress resilience and anabolic balance.
• Testosterone–An indicator of physiological adaptation and recovery capacity.

#### 2.4.2 Blood analysis

• Hormone quantification was performed using enzyme-linked immunosorbent assays (ELISA) in a certified clinical laboratory.
• Results were analyzed to determine pre-vs post-hypoxia differences, allowing for a direct assessment of hypoxia’s impact on endocrine regulation in aviation professionals.

### 2.5 Statistical analysis

The data were analyzed using IBM SPSS Statistics software (Version 24.0). Descriptive statistics were calculated for each variable, with data presented as Mean ± Standard Deviation (SD). Repeated measures ANOVA and post-hoc Fisher LSD test were used. A significance level of p < 0.05 was set for all analyses to determine statistical significance. The effect sizes (Cohen’s d) were calculated to assess the magnitude of change in the intervention group, particularly to evaluate the practical significance of IHT on cognitive and physiological outcomes. Additionally, changes between pre- and post-intervention values were illustrated to visualize trends across the different variables analyzed, providing a clear interpretation of the impact of IHT on psychophysiological resilience.

## 3 Results

Participants’ characteristics, divided by pilots and flight attendants, are detailed in Tables 1, 2. The average peripheral oxygen saturation (SpO_2_) values during hypoxia exposure were 93.6% ± 1.35% for pilots and 93.4% ± 1.50% for flight attendants 1 hour after the start of exposure. These levels increased slightly to 94.8% ± 1.29% for pilots and 94.6% ± 1.42% for flight attendants due to ventilatory adaptations but dropped to 93.2% ± 1.58% for pilots and 92.9% ± 1.68% for flight attendants the following morning. This suggests a slightly faster adaptation among pilots, possibly related to their higher baseline oxygen efficiency from operating under cabin pressure conditions during flights.

**TABLE 1:** Procedure.

Day	Time	Activities	Hypoxia exposure	Key notes
Day 1	Blood work at 9:00 a.m.	Participants followed their typical workday routine, including piloting private flights of 3–4 h duration. Only low-intensity physical activities were performed	None	Baseline data collected under normal occupational conditions
Day 2	No blood work	Regular work schedule with flights of 3–4 h duration. Participants adhered to consistent dietary intake and physical activity levels	None	Ensured consistency in external factors such as diet, physical activity, and rest
Day 3	Blood work at 9:00 a.m.; Hypoxia from 8:00 p.m.–8:00 a.m.	Blood work collected prior to the intervention. Hypoxia exposure initiated at night using MITOVIC® mask system, simulating an altitude of 3,500–4,000 m	First hypoxia session: Simulated altitude of 3,500–4,000 m (FiO_2_ ∼14.5%) via MITOVIC® system	Monitored SpO_2_ levels at multiple time points during the hypoxia exposure
Day 4	Blood work at 9:00 a.m.; Hypoxia from 8:00 p.m.–8:00 a.m.	Participants refrained from any work activities, focusing on rest. Second hypoxia session conducted under identical conditions as Day 3	Second hypoxia session: Simulated altitude of 3,500–4,000 m (FiO_2_ ∼14.5%) via MITOVIC® system	Data collected for the experimental period. Ensured optimal conditions for hypoxia adaptation and recovery
Day 5	Blood work at 9:00 a.m.	Participants returned to their typical work schedule, including private flights of 3–4 h duration. No additional hypoxia exposure occurred	None	Final blood sampling conducted post-intervention under fasting conditions to assess hormonal changes

This table presents the procedure followed throughout the study by the patients.

**TABLE 2:** Participants’ characteristics by group.

Variable	Group	Mean	Min	Max	SD
Height (cm)	Pilots	181.25	172.00	189.00	6.584
	Flight Attendants	171.45	165.00	177.00	4.935
Weight (kg)	Pilots	82.15	70.00	92.00	7.483
	Flight Attendants	67.85	60.00	78.00	5.934
BMI (kg/m^2^)	Pilots	24.99	22.01	27.89	2.139
	Flight Attendants	23.12	20.95	26.30	1.754

Table 3 presents changes in hormone levels divided by groups. Depicting values as mean ± SD.

**TABLE 3:** Changes in hormone levels by group.

Hormone	Group	Baseline 1 (mean ± SD)	Baseline 2 (mean ± SD)	Post-intervention (mean ± SD)
Prolactin (mIU/L)	Pilots	225.8 ± 117.5	220.6 ± 62.0	376.8 ± 112.5
	Flight Attendants	210.2 ± 119.3	205.7 ± 61.8	360.5 ± 110.8
Testosterone (nmol/L)	Pilots	22.6 ± 6.45	22.7 ± 5.85	24.7 ± 5.55
	Flight Attendants	1.95 ± 0.48	2.00 ± 0.50	2.30 ± 0.55
Cortisol (nmol/L)	Pilots	370.2 ± 112.8	378.5 ± 104.7	450.6 ± 70.2
	Flight Attendants	360.5 ± 111.2	368.2 ± 103.4	440.3 ± 68.5
DHEA-S (μmol/L)	Pilots	8.70 ± 4.12	8.75 ± 3.65	8.95 ± 3.88
	Flight Attendants	8.10 ± 4.18	8.20 ± 3.72	8.45 ± 3.90
DHEA-S/Cortisol Ratio	Pilots	0.023 ± 0.009	0.023 ± 0.009	0.018 ± 0.006
	Flight Attendants	0.022 ± 0.008	0.022 ± 0.008	0.017 ± 0.007

Data is expressed as mean ± SD. DHEA-S: dehydroepiandrosterone sulfate.

**FIGURE 1:**
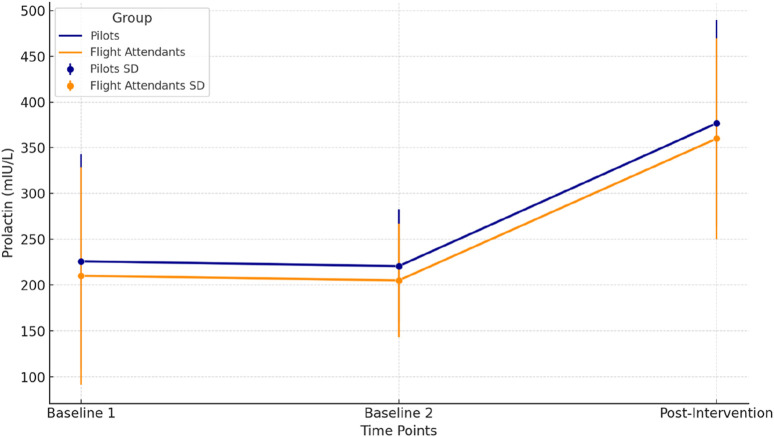
Prolactin levels across time points for pilots and flight attendants.

Figure 1 illustrates the changes in prolactin levels (mIU/L) across three time points (Baseline 1, Baseline 2, and Post-Intervention) for pilots (dark blue) and flight attendants (dark orange). Each line represents the mean prolactin levels, and the error bars indicate the standard deviation (SD) at each time point. In pilots, prolactin levels remained stable between Baseline 1 and Baseline 2 but showed a significant increase after the intervention, reflecting an acute stress response to hypoxia. In flight attendants, a similar trend was observed, with levels increasing significantly after the intervention, though at slightly lower absolute values compared to pilots. Significant changes were identified using repeated measures ANOVA and post-hoc Fisher LSD tests Significant increase post-intervention (pilots p = 0.031; flight attendant p = 0.028), determined via repeated measures ANOVA followed by Fisher’s LSD post-hoc test.

**FIGURE 2:**
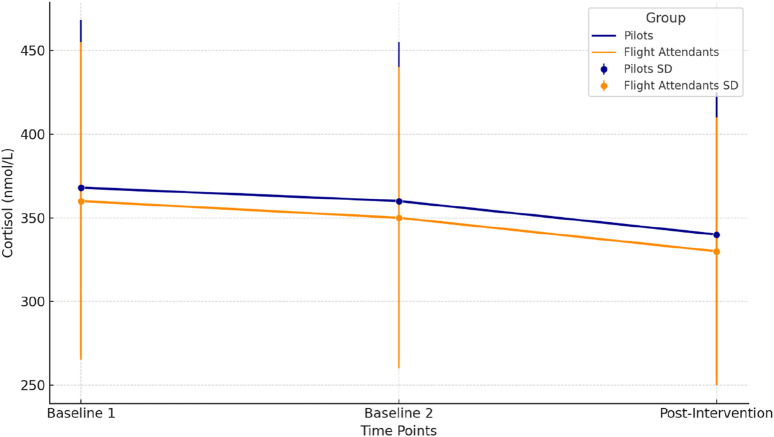
Cortisol levels across time points for pilots and flight attendants.

Figure 2 represents the variations in cortisol levels (nmol/L) across three time points (Baseline 1, Baseline 2, and Post-Intervention) for pilots (dark blue) and flight attendants (dark orange). Each line illustrates the mean cortisol levels for the respective group, with error bars indicating the standard deviation (SD). Pilots present a slight but consistent decrease in cortisol levels was observed from Baseline 1 to Post-Intervention, reflecting a physiological recovery following the hypoxia protocol. Flight Attendants are similar to the pilots, cortisol levels showed a gradual decline, suggesting a comparable recovery trend in this group. Cortisol levels decreased post-intervention in both groups. Significant changes were identified, Pilots experienced a significant decrease in cortisol levels (p = 0.034). A significant reduction was also observed (p = 0.028) in Flight Attendants, determined via repeated measures ANOVA followed by Fisher’s LSD post-hoc test.

**FIGURE 3:**
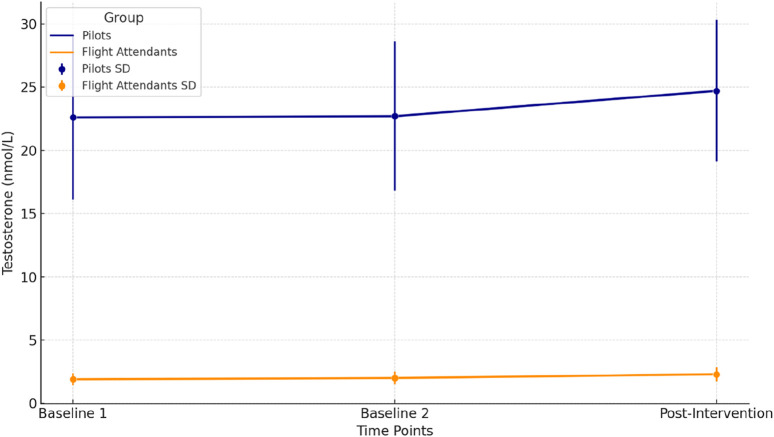
Testosterone levels across time points for pilots and flight attendants.

Figure 3 displays the changes in testosterone levels (nmol/L) across three time points (Baseline 1, Baseline 2, and Post-Intervention) for pilots (dark blue) and flight attendants (dark orange). The mean values are shown as lines, and the error bars represent the standard deviation (SD). Testosterone levels for pilots show a slight increase from Baseline 1 to Post-Intervention, reflecting a physiological response to the hypoxia intervention. Also, Flight Attendants’ testosterone levels exhibit a small but consistent increase across time points, aligning with the general trend observed in pilots. Pilots’ increase was significant post-intervention (p = 0.041). In Flight Attendants, the increase was marginally significant (p = 0.049), determined via repeated measures ANOVA followed by Fisher’s LSD post-hoc test.

**FIGURE 4:**
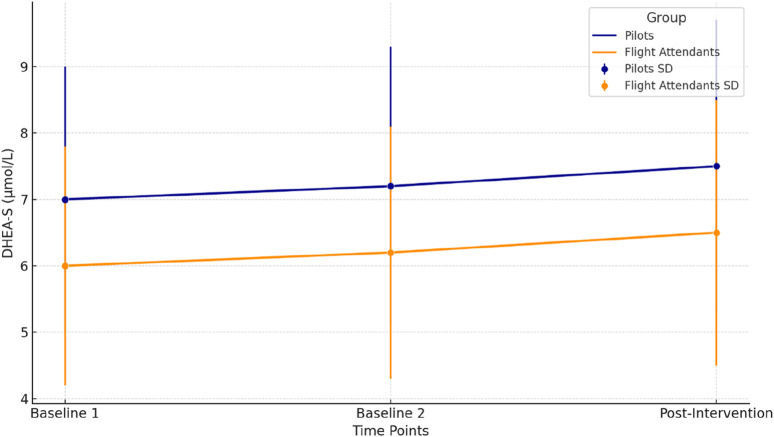
DHEA-S levels across time points for pilots and flight attendants.

Figure 4 represents the changes in DHEA-S levels (μmol/L) across three time points (Baseline 1, Baseline 2, and Post-Intervention) for pilots (dark blue) and flight attendants (dark orange). The mean values are plotted as lines, with error bars indicating the standard deviation (SD) for each time point. Pilots present a gradual increase in DHEA-S levels was observed across all time points, suggesting a positive adaptation to the hypoxia intervention. Similarly, a slight increase in DHEA-S levels was noted in Flight Attendants, reflecting a comparable trend of anabolic response. Further on, Pilots presented a significant increase in post-intervention (p = 0.039). However in Flight Attendants, the increase was marginally significant (p = 0.042), determined via repeated measures ANOVA followed by Fisher’s LSD post-hoc test.

**FIGURE 5:**
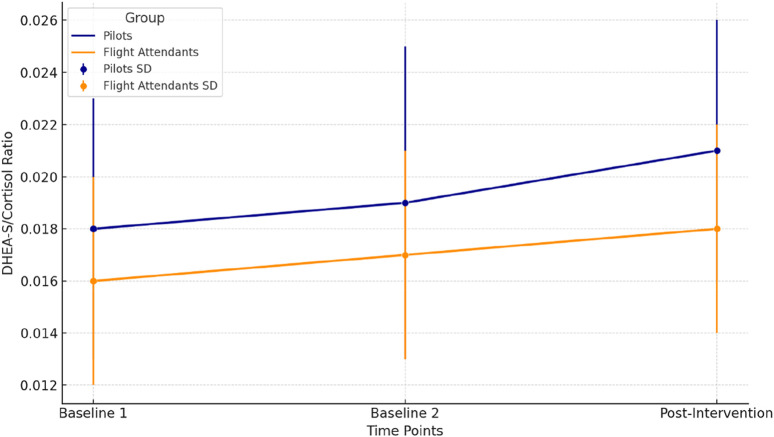
DHEA-S/cortisol ratio across time points for pilots and flight attendants.

Figure 5 presents the changes in the DHEA-S/Cortisol ratio across three time points (Baseline 1, Baseline 2, and Post-Intervention) for pilots (dark blue) and flight attendants (dark orange). The mean values are displayed as lines, and error bars indicate the standard deviation (SD) for each time point. Pilots present a progressive increase in the ratio observed, reflecting a shift towards an anabolic state, which could indicate enhanced stress recovery after the hypoxia intervention. Flight Attendants present a similar upward trend is seen, although the absolute values remain slightly lower compared to pilots, suggesting a comparable but less pronounced adaptation. The increase was significant post-intervention in pilots (p = 0.035) and flight attendants (p = 0.039), determined via repeated measures ANOVA followed by Fisher’s LSD post-hoc test.

The analysis of HRV metrics following the hypoxia training intervention showed significant improvements in key markers of autonomic regulation. The mean HR decreased significantly from 68.5 ± 9.5 bpm at baseline to 63.2 ± 10.3 bpm post-intervention. This reduction in HR suggests improved autonomic balance and cardiovascular efficiency due to the hypoxia training. The RR interval, representing the time between heartbeats, also showed a significant decrease from 870.2 ± 130.5 ms to 820.4 ± 120.7 ms, indicating a faster recovery rate.

Measures of short-term HRV, such as pNN50 and RMSSD, demonstrated improvements in the intervention group, though to varying degrees. The pNN50, which reflects the percentage of successive RR intervals that differ by more than 50 ms, decreased from 18.7 ± 16.5 to 12.3 ± 11.8. RMSSD, indicative of parasympathetic activity, reduced from 43.0 ± 22.3 ms to 35.8 ± 20.5 ms. These changes suggest enhanced autonomic flexibility, with the intervention adapting to stressors, which did not exhibit significant changes in these parameters. SDNN, a marker of overall HRV, decreased slightly in the intervention from 52.3 ± 24.8 ms to 48.1 ± 20.1 ms, reflecting changes in overall autonomic function. The control group’s SDNN remained largely unchanged, further emphasizing the influence of hypoxia training on autonomic responses.

Frequency-domain measures of HRV, including HF and low-frequency LF components, revealed noteworthy trends. HF power, associated with parasympathetic activity, decreased in the intervention from 36.5 ± 15.7 ms^2^ to 28.0 ± 13.5 ms^2^, while LF power, reflecting both sympathetic and parasympathetic contributions, increased from 63.5 ± 17.5 ms^2^ to 72.0 ± 15 ms^2^. The LF/HF ratio, an indicator of sympathetic to parasympathetic balance, increased from 2.8 ± 2.1 to 3.4 ± 1.8 in the intervention group, suggesting a shift towards greater sympathetic activation post-intervention, which is typically observed with enhanced physical readiness. Other HRV parameters, such as total power and geometric measures like SD1 and SD2, also demonstrated significant differences in post-intervention. Total power, which captures overall HRV, decreased in the intervention from 2,750.5 ± 370.2 ms^2^ to 2,250.7 ± 320.6 ms^2^. SD1, which reflects short-term variability, reduced from 30.1 ± 18.5 ms to 25.3 ± 16.7 ms, while SD2, related to long-term variability, decreased from 62.8 ± 31.2 ms to 58.0 ± 28.9 ms.

## 4 Discussion

Stress is a significant physiological and psychological burden, particularly for professionals working in high-demand environments such as private aviation pilots and flight attendants (Folke and Melin, 2024). These individuals are subjected to prolonged work hours, rapid decision-making under pressure, frequent time zone shifts, and constant exposure to altered atmospheric conditions, particularly hypoxia due to cabin pressurization (Leatherwood and Dragoo, 2013). Over time, chronic exposure to these stressors can contribute to dysregulation of the hypothalamic-pituitary-adrenal (HPA) axis, increased risk of cardiovascular disease, impaired cognitive function, and a compromised immune response (Young et al., 2021). Given the substantial physiological demands of their profession, effective interventions that support recovery and resilience are essential. Controlled hypoxia exposure has been proposed as a potential strategy to enhance stress recovery by modulating endocrine function and improving overall wellbeing. Our study aimed to evaluate the effects of a short-term normobaric hypoxia intervention on key hormonal markers, including cortisol, prolactin, testosterone, and DHEA-S, in private aviation professionals. The results revealed significant alterations in these biomarkers, suggesting that hypoxia exposure during rest periods may serve as an effective recovery tool for individuals in high-stress occupations.

One of the most notable findings was the significant increase in prolactin levels post-intervention in both pilots and flight attendants. Prolactin is recognized as a neuroendocrine stress marker, with elevations often associated with both acute and chronic stress conditions (Lennartsson and Jonsdottir, 2011). This is consistent with previous research on individuals exposed to high-altitude environments, where increased prolactin secretion has been attributed to the body’s adaptation to lower oxygen availability. Gonzales et al. (2011) reported a significant rise in prolactin following high-altitude hypoxia exposure, likely due to increased hypothalamic activation in response to environmental stress. Our findings align with this hypothesis, suggesting that even controlled normobaric hypoxia is sufficient to elicit a measurable neuroendocrine response. Interestingly, while both pilots and flight attendants exhibited this increase, the magnitude of change was slightly greater in pilots, potentially due to differences in baseline stress load or gender-based variations in prolactin regulation. Prior research indicates that females generally exhibit lower prolactin responses to hypoxia, possibly due to interactions with testosterone and differences in dopaminergic regulation (Alcantara-Zapata et al., 2023; Lennartsson and Jonsdottir, 2011). The use of two 12-h overnight hypoxia sessions was purposefully chosen to evaluate the acute hormonal response to a feasible, time-limited intervention that aligns with aviation professionals’ mandatory rest periods. Rather than attempting to induce long-term adaptations, the protocol aimed to simulate a practical recovery strategy that could be applied in real-world occupational settings. This approach reflects the operational constraints of aviation personnel, where extended training periods are often unfeasible due to flight schedules and regulatory limits.

Cortisol, a central regulator of the HPA axis and a primary stress hormone, exhibited a moderate decrease post-intervention. This result diverges from studies that have documented cortisol elevations following hypoxia exposure, particularly under acute or severe hypoxic stress conditions. Individuals exposed to altitudes above 4,000 m typically experience significant cortisol increases, reflecting a stress response aimed at mobilizing energy reserves (Mazzeo, 2008). However, the design of our study differed in several key aspects. The controlled nature of our hypoxia intervention, coupled with its incorporation into the participants’ designated rest period, may have attenuated the typical stress response, facilitating physiological adaptation rather than triggering acute stress. Additionally, chronic occupational stress is known to induce HPA axis dysregulation, which can alter cortisol responsiveness to new stressors (Young et al., 2021). Given the prolonged exposure of aviation professionals to work-related stress, their baseline cortisol regulation may differ from that of the general population, potentially explaining the observed decrease in post-intervention levels.

Testosterone levels exhibited a significant increase following the intervention, further supporting the hypothesis that controlled hypoxia may enhance anabolic recovery mechanisms. Previous studies have demonstrated that high-altitude exposure can stimulate testosterone production, particularly in endurance-trained athletes undergoing altitude training. Beall et al. (2003) found that hypoxia-induced erythropoietin (EPO) production enhances testosterone synthesis through downstream metabolic pathways. Our findings indicate that even short-term normobaric hypoxia can provide a stimulus for anabolic adaptation, as both pilots and flight attendants exhibited increased testosterone levels post-intervention. This has important implications for aviation professionals, as testosterone plays a critical role in muscle repair, cognitive function, and stress resilience. Higher testosterone levels have been associated with improved executive function and decision-making, which are particularly relevant for individuals working in high-pressure environments. Additionally, our results align with those of Millet et al. (2010), who demonstrated that hypoxic conditioning can enhance both physical and mental performance through its effects on anabolic hormones. The greater testosterone response observed in pilots compared to flight attendants is likely attributable to baseline gender differences, as males naturally produce higher testosterone levels and tend to exhibit a more pronounced response to anabolic stimuli.

DHEA-S, an androgen precursor with well-documented anti-inflammatory and neuroprotective properties, exhibited a slight but non-significant increase post-intervention. DHEA-S is recognized for its role in counteracting cortisol’s catabolic effects and promoting resilience to chronic stress. Shields et al. (2016) noted that higher DHEA-S levels are associated with better cognitive function and reduced susceptibility to stress-related disorders. Although the observed increase in DHEA-S in our study did not reach statistical significance, the trend suggests that repeated or prolonged hypoxia exposure could amplify this response over time. More importantly, the DHEA-S/cortisol ratio—a key indicator of the body’s stress adaptation capacity—showed a favorable shift post-intervention. This finding is particularly relevant, as a higher DHEA-S/cortisol ratio has been linked to improved recovery from stress and greater psychological resilience. Dutheil et al. (2021) emphasized the importance of maintaining a balanced DHEA-S/cortisol ratio in individuals exposed to chronic occupational stress, as disruptions in this balance have been associated with burnout, cognitive decline, and mood disorders.

A key aspect of our findings is the similarity in hormonal responses between pilots and flight attendants, despite baseline gender differences in hormone levels. While males typically exhibit higher levels of testosterone and cortisol, and females tend to have higher baseline prolactin, the overall trends post-intervention were consistent across both groups. This suggests that controlled hypoxia may provide universal benefits for stress recovery and adaptation, regardless of gender. However, the magnitude of change was generally greater in pilots, possibly reflecting differences in baseline stress exposure, workload, or physiological stress sensitivity.

The physiological mechanisms underlying these hormonal adaptations can be attributed to the interplay between hypoxia-inducible factor-1 alpha (HIF-1α) and the HPA axis (Vanderhaeghen, Beyaert, and Libert, 2021). HIF-1α, a transcription factor activated under hypoxic conditions, regulates numerous genes involved in oxygen transport, angiogenesis, and metabolic adaptation (Ziello, Jovin, and Huang, 2007). HIF-1α activation has been shown to influence cortisol secretion and may also play a role in the prolactin and testosterone responses observed in our study. Additionally, hypoxia-induced EPO production could contribute to the anabolic effects seen in testosterone elevation. The interaction between these pathways highlights the potential of hypoxia as a physiological stressor that can be harnessed for recovery and performance optimization.

From a practical perspective, our findings suggest that integrating controlled hypoxia sessions into structured rest periods may serve as an effective strategy for mitigating the effects of chronic stress in aviation professionals. By modulating stress hormones and promoting an anabolic hormonal profile, this approach may enhance recovery, improve cognitive performance, and reduce long-term health risks associated with occupational stress. These findings have important implications not only for aviation but also for other high-stress professions, such as emergency responders, military personnel, and elite athletes. However, further research is needed to refine optimal hypoxia protocols, determine long-term effects, and assess individual variability in response.

Several limitations should be acknowledged. The relatively small sample size limits the generalizability of our findings, and the absence of subjective stress assessments prevents a direct correlation between hormonal changes and perceived wellbeing. Future studies should incorporate larger cohorts, longer follow-up periods, and subjective stress measures to provide a more comprehensive understanding of the relationship between hypoxia exposure and occupational stress recovery.

In conclusion, our study provides evidence that short-term controlled normobaric hypoxia can induce significant hormonal adaptations in private aviation professionals. The observed changes in prolactin, cortisol, testosterone, and the DHEA-S/cortisol ratio highlight the potential of hypoxia as a tool for stress recovery and resilience. Integrating hypoxia exposure into structured recovery protocols may help aviation professionals better adapt to chronic occupational stress, ultimately enhancing their health, performance, and overall wellbeing.

While the hormonal changes observed in this study—such as increased prolactin and testosterone levels—are statistically significant and indicative of physiological adaptation to hypoxia, they should be interpreted as functional, rather than clinically diagnostic, shifts. The values remained within normal physiological ranges for healthy adults, and no participant exhibited symptoms suggestive of hormonal imbalance. Therefore, these results likely reflect adaptive neuroendocrine modulation rather than changes with direct clinical relevance. Future studies with longitudinal follow-up and clinical assessments would be necessary to determine whether such responses could contribute to long-term health or performance outcomes.

### 4.1 Practical applications

The findings of this study have significant implications for the occupational health of aviation professionals, particularly private pilots and flight attendants, who experience high levels of cumulative stress due to irregular schedules, sleep disruption, and frequent exposure to altitude-related physiological challenges. The observed hormonal changes suggest that short-term exposure to normobaric hypoxia during rest periods may serve as an effective tool for modulating stress hormones and enhancing recovery. This approach could be integrated into structured recovery programs, offering a non-pharmacological method to improve endocrine balance, mental clarity, and physiological resilience. Given the observed increase in testosterone and the favorable shift in the DHEA-S/cortisol ratio, this intervention could also have potential applications in optimizing cognitive function and physical performance, both of which are critical for aviation professionals responsible for high-stakes decision-making.

Future research should aim to refine and optimize the parameters of hypoxia exposure, investigating the effects of different hypoxia intensities, durations, and frequencies. The potential benefits of intermittent hypoxia training (IHT) should be further explored, particularly in relation to its ability to improve stress adaptation and long-term hormonal balance. Additionally, comparative studies examining the effects of normobaric versus hypobaric hypoxia on stress biomarkers could provide valuable insights into the most effective recovery strategies. Longitudinal studies should also assess whether repeated exposure to hypoxia over extended periods leads to cumulative benefits in stress resilience, cardiovascular health, and overall wellbeing.

Beyond aviation, these findings could be applied to other high-stress professions, such as emergency responders, military personnel, and elite athletes. The potential for controlled hypoxia to act as a pre-conditioning stimulus for individuals preparing for high-altitude work or operations should also be examined, as it may facilitate better physiological adaptation and reduce the adverse effects of altitude sickness. Furthermore, research into the psychological aspects of hypoxia exposure, including its effects on perceived stress and mood regulation, could enhance our understanding of the broader applications of this intervention in mental health and stress management programs.

### 4.2 Limits of the study

While this study provides valuable insights into the hormonal responses to normobaric hypoxia in aviation professionals, several limitations must be acknowledged. First, the study included only pre-menopausal women and middle-aged males, which may limit the applicability of the findings to other age groups, such as post-menopausal women or older individuals. Hormonal responses to hypoxia can be influenced by age-related endocrine changes, particularly in women, where menopausal status can significantly affect hormonal profiles. Furthermore, hormonal responses to hypoxia may differ according to sex and physical training background. Our sample consisted of actively working aviation professionals with a relatively homogeneous occupational profile, and we did not include a control group from other professions or populations with varying levels of physical conditioning. Future studies should aim to include larger cohorts to ensure greater statistical power and broader applicability of the results. Second, although the hormonal changes observed provide objective evidence of physiological adaptation, the study did not incorporate subjective measures of stress perception, fatigue, or wellbeing. Including validated psychological scales, such as the Perceived Stress Scale (PSS) or the Profile of Mood States (POMS), would allow for a more comprehensive assessment of the intervention’s impact on both physiological and psychological stress markers.

Additionally, while we observed significant endocrine changes, the study did not assess potential behavioral or cognitive effects associated with these hormonal shifts. Given the established link between testosterone and cognitive function, future research should include neurocognitive assessments to determine whether hypoxia exposure translates into measurable improvements in reaction time, attention, and decision-making under stress. Another limitation is that the study only examined the acute effects of a single weekend of hypoxia exposure. It remains unclear whether these hormonal changes persist over time or if repeated exposure to hypoxia would lead to cumulative benefits. Long-term follow-up studies are necessary to assess the duration and sustainability of these adaptations. Furthermore, no follow-up hormonal measurements were conducted beyond the immediate post-intervention period. This prevents us from determining the duration or stability of the observed hormonal changes. Future studies should include delayed follow-up time points, ideally ranging from several days to 1 month post-intervention, to evaluate whether the endocrine responses are transient or indicative of longer-term adaptation.

Finally, the study was conducted in a controlled environment, minimizing confounding variables but potentially limiting ecological validity. While this controlled setting was necessary to isolate the effects of hypoxia, real-world applications may involve additional variables, such as workload, flight duration, and environmental stressors, which could influence the outcomes. Future studies should explore how hypoxia exposure interacts with these occupational stressors in actual aviation settings.

Another important limitation is the absence of a control group or sham-hypoxia condition. While the within-subject design allows for the detection of time-based changes, it limits our ability to draw definitive causal inferences regarding the specific effects of hypoxia exposure. Without a comparator group, we cannot exclude the possibility that some of the observed changes may be attributable to circadian fluctuations, placebo effects, or other unmeasured variables. Future studies should incorporate randomized controlled designs, ideally including a placebo or sham-hypoxia condition, to confirm that the hormonal and autonomic responses are specifically attributable to the hypoxic stimulus.

## 5 Conclusion

This study provides evidence that short-term exposure to normobaric hypoxia, when integrated into a structured recovery period, can elicit significant hormonal adaptations in private aviation professionals. The observed increases in prolactin and testosterone, along with the favorable shift in the DHEA-S/cortisol ratio, suggest that controlled hypoxia may serve as a valuable intervention for modulating stress hormones and enhancing physiological resilience. The reduction in cortisol levels, contrary to findings in acute hypoxic stress studies, highlights the potential for hypoxia to function as a recovery aid rather than merely a stressor.

The findings support the idea that structured hypoxia exposure could be integrated into occupational health programs aimed at mitigating the long-term effects of chronic stress in aviation professionals. By leveraging hypoxia as a physiological conditioning tool, pilots and flight attendants may improve their hormonal balance, cognitive function, and overall wellbeing, ultimately enhancing their capacity to perform under high-pressure conditions.

Future research should expand on these findings by exploring different hypoxia protocols, assessing long-term adaptation, and incorporating neurocognitive and psychological measures. The potential for hypoxia to serve as a pre-conditioning tool for high-altitude work and extreme occupational environments should also be investigated. While the current findings provide a promising foundation, additional research is needed to refine and optimize hypoxia-based recovery strategies, ensuring their efficacy and safety for long-term implementation in occupational and clinical settings.

## Data Availability

The original contributions presented in the study are included in the article/supplementary material, further inquiries can be directed to the corresponding author.
